# Obstacles to intergenerational communication in caregivers’ narratives regarding young people’s sexual and reproductive health and lifestyle in rural South Africa

**DOI:** 10.1186/s12889-020-08780-9

**Published:** 2020-05-27

**Authors:** Bo Nilsson, Kerstin Edin, John Kinsman, Kathleen Kahn, Shane A. Norris

**Affiliations:** 1 Department of Culture and Media Studies, Umeå University, Umeå, Sweden; 2 Department of Nursing, Sexual and Reproductive Health, Umeå University, Umeå, Sweden; 3 Department of Epidemiology and Global Health, Umeå University, Umeå, Sweden; 4 MRC/Wits Rural Public Health & Health Transitions Research Unit (Agincourt), School of Public Health, Faculty of Health Sciences, University of the Witwatersrand, Johannesburg, South Africa; 5 Department of Public Health Sciences, Global Health (IHCAR), Karolinska Institutet, Stockholm, Sweden; 6 INDEPTH Network, Accra, Ghana; 7 MRC/Wits Developmental Pathways for Health Research Unit, Faculty of Health Sciences, University of the Witwatersrand, Johannesburg, South Africa

**Keywords:** Sexual and reproductive health, Respectability, Discourse theory, Moral regime, Intergenerational communication

## Abstract

**Background:**

Statistics from South Africa show the world’s highest HIV prevalence with an estimated seven million people living with the virus. Several studies have pointed to communication about sexuality between parents/caregivers and children as a protective factor. However, communication between generations has been described as problematic, especially due to discomfort in discussing sexual matters. The aim of this study was to explore how caregivers in a poor, rural part of South Africa talked about young people in general, their sexuality, and their lifestyle practices. A particular interest was directed towards central discourses in the caregivers’ narratives and how these discourses were of importance for the caregivers to function as conversation partners for young people.

**Methods:**

In this qualitative study convenience sampling was used to select and invite participants. Information was collected from nine one-on-one interviews conducted with caregivers from rural areas within South Africa. The interview guide included nine main questions and optional probing questions. Each interview took place in an uninterrupted setting of choice associated with the caregivers’ home environment. The interviews were transcribed and analyzed using discourse analysis.

**Results:**

Interview narratives were characterized by three central discourses – *demoralized youths in a changing society*, *prevailing risks and modernity* and *a generation gap.* The *youths* were discursively constructed *as a problematic group* relating to specific prevailing risks such as *early pregnancies*, *modern technologies*, *STI/HIV* and *contraceptives*. The interview narratives illustrated that caregivers tried to impose their views of a *respectable* lifestyle in young people. At the same time caregivers expressed a *morality of despair* mirroring a generation gap which counteracted their ability to communicate with their children and grandchildren.

**Conclusions:**

The findings add to the body of earlier research illustrating that rural South African caregivers and their children/grandchildren hold different moral standards. The interview material reflected a ‘clash’ between generations relating to their differing perceptions of a desirable lifestyle. To overcome the generational gap, we recommend further research about how a well-founded national and community collaboration linked to school-based programs can support family participation in order to empower adults in their communication with young people.

## Background

Young people are exposed to sexual and reproductive health risks when having sex without adequate contraception and protection, leading to an increased probability of unintended pregnancies and sexually transmitted infections (STIs), including human immunodeficiency virus (HIV). The explanations for people’s unsafe sexual behaviors are diverse and multifaceted with many societal, cultural, and individual reasons [1]. The highest rates of adolescent pregnancies (age 15–19) occur in low-income countries [2], and such pregnancies are largely associated with low socio-economic status, poor school achievement, and risky behaviors and are often seen from one generation to the next [3].

Statistics from South Africa (SA) show the world’s highest HIV prevalence with an estimated seven million people living with HIV, which represent 19% of the adult population [4]. The results from a survey in 2012 showed that young people aged 15–24 years accounted for 25% of newly HIV-infected people in SA [5]. The HIV prevalence in the country for young women (aged 15–24 years) is as much as four times higher compared to young men in the same age group [5]. The disparity between men and women in terms of HIV prevalence has numerous explanations. Normative gender patterns often result in the peer pressure of dating and having sex as compulsory, while trivializing the risk of unintended pregnancies and STIs, including HIV, when using no protection [6]. Several studies describe how a considerable number of young women experience gender inequality with subordination and dependency when involved in forced or transactional sex and hence have limited decision-making opportunities in order to protect themselves [7–10]. Moreover, poverty makes the situation for women even worse regarding low status and dependency on men, especially when it comes to negotiation for safe sex or to resisting forced sex, violence, and age-disparate sexual contacts [11, 12].

High rates of unsafe sex and sexual risk taking have, for several decades, been seen to relate to SA as a society characterized by different moral regimes with a clash between older and younger generations [13, 14]. According to Harrison [15], young people regard open expressions of sexuality as ‘modern’, while their parents usually oppose sexual activities before marriage and expect ‘good behavior’, especially for teenage girls, and believe in ‘traditional’ moral regimes according to which marriage and reproduction are central [16]. Young people therefore try to hide sexual activities from adults because they are aware of the possible dishonor they can bring, while at the same time they embrace romantic love with sex as being interconnected and as a part of becoming grown-up [17].

Several studies have pointed out communication about sexuality between parents/caregivers and children as a protective factor for sexual behavior [18, 19]. Moreover, interventions on sexual and reproductive health issues are supposed to have a better outcome when including not only children, but also their caregivers and families [2, 20]. However, communication between generations has been described as problematic, especially due to discomfort in discussing sexual matters [18, 21] or a fear among parents of being too strict towards their children [22]. A review of research conducted in sub-Saharan Africa pointed out that parents (in general) are often regarded as playing an important role in sexual and gender socialization, but that they face many barriers in this such as a historical taboo against authoritarian manners regarding sexuality communication, as well as the challenge that discussions about sexuality may often turn into an intangible and implicit one-way communication [23]. A study from Tanzania showed that one good way to promote healthy sexual behavior is for parents to spend time together with their children and thereby enable a sense of connectedness and parent-child communication [24]. Communication between adults and young people has, according to research from SA, been described as inadequate [25], which might therefore miss opportunities to talk about minimizing unsafe sexual behavior [26]. In a previous paper [6], we presented results from interviews with young men and women in the same area of SA as studied in this paper. The young interviewees mentioned how difficult it was to be straightforward in communication about sensitive and personal issues with both caregivers and teachers (despite the school subject Life Orientation), and they therefore preferred to talk to friends [6]. We interpreted this difficulty as a gap between generations and as an important theme to investigate further. In this study we therefore explore discourses in caregivers’ narratives of young people in a poor and rural part of SA, and how these discourses were of importance for the caregivers to function as role models.

The aim of this study was to explore how caregivers in a poor rural part of SA talked about young people in general, their sexuality, and their lifestyle practices. A particular interest was directed towards central discourses in the caregivers’ narratives and how these discourses were of importance for the caregivers to function as conversation partners for young people.

### Theoretical framework

Discourses are central for how people think about topics, what they value, and how they behave [27]. Discourse theory as presented by Laclau and Mouffe [28] can be understood as a strategy for analyzing the ‘fixation of meaning’ in terms of discourses. Discourses regulate what is meaningful to talk about and how to talk about it. However, the fixation of meaning process will never be completed because there will always be competitions between different discourses regarding the establishment of a hegemonic ‘world view’. In this study, we have identified three central discourses from the caregivers’ narratives – *demoralized youths in a changing society*, *prevailing risks and modernity and a generation gap.*

‘Moral regime’ can be defined as a system of meanings that regulates what is regarded to be normal, decent, and desirable behavior, and what is considered to be the opposite – deviant, inappropriate, and undesirable behavior. However, a moral regime is not only active on a cognitive level; it includes power and controls individual behaviors and social relations in practice [29]. Furthermore, a moral regime can have its own unique norms and rules [14] and be organized around central notions. In this study, ‘ambivalence’ and ‘respectability’ [30] are two central – and partly contradictory – notions in a regime we call ‘morality of despair’. This refers to caregivers’ feelings of powerlessness and despair in talking about their children and grandchildren as well as young people in general. Specifically, morality of despair is in this study characterized by caregivers’ worries about how to raise their children and grandchildren to be moral and respectable adults.

## Methods

### Design

A qualitative design, based on nine individual interviews with caregivers in a rural part of SA, was used for this study.

### Setting

This study was part of a larger research project [6, 31] carried out in Agincourt sub-district of Bushbuckridge, Mpumalanga province, in northeast SA. Agincourt is a rural, and economically disadvantaged area, characterized by high rates of unemployment, work-related migration and many households are reliant on social grants. The MRC/Wits Rural Public Health and Health Transitions Research Unit has been running a health and socio-demographic surveillance system (HDSS) in this site since 1992, where the locals are accustomed to ongoing research activities. The study area covers 420 km^2^ and included 27 villages and around 90,000 inhabitants during the time of the study [31, 32]. In Agincourt, many young people are at risk of becoming parents at an early age and to contract sexually transmitted infections including HIV [31]. The HIV prevalence for people in Agincourt aged 15 years and above was 19,4% (10,6% for men and 23,9% for women) in 2011 [33].

### Participants

To receive approval for our study, the second author contacted PEO (MRC/Wits Research Unit’s Public Engagement Office). The Office facilitated communication between the research team, village leaders and village communities, and assumed responsibility for local dissemination of the research findings. The participants, who were well informed about the ongoing work of MRC/Wits Research Unit, comprised nine caregivers (one man and eight women). Seven were around 40–50 years old and two were grandmothers aged over 60 years old. The interviews were conducted in February and March 2012.

### Sampling method

This study was part of a larger research project, in which a random sample of 30 young men and women (aged 18–19) and young mothers (≤19 years old when they gave birth) were, through the HDSS database, identified in three selected villages [6, 31]. When doing home-visits for interviews with the young participants in the three villages [6], we used convenient sampling to also recruit present caregivers. The first one at each household who agreed to be interviewed was included. Finally, nine caregivers approved and none of them dropped out.

### Data collection method

Data was collected from in-depth interviews. The three local women who carried out the interviews were professional fieldworkers with prior experience from research projects. They were employed by MRC/Wits Research Unit and trained in qualitative methods. Before starting this project, all fieldworkers attended a three-day training session conducted by the second author. The research team had no personal interest in the study nor personal contact with the participants prior to the interviews.

A semi-structured interview guide was used, which included nine common questions, and additionally between one and three probing questions with the objective to elicit more in-depth responses. The guide was culturally adapted after input from local fieldworkers, and translated into Shangaan (the local language). Each one-on-one interview took place in an undisturbed and private setting of choice, e.g. in the participants’ home or in their garden.

The questions related mainly to lifestyle and early pregnancies among young people. The second author (KE) was not attending but was present during all the interviews and took part in daily debriefing sessions (in English) during which time the data collection team held discussions and made short field notes about the content and quality of interviews. Eight interviews were audio recorded digitally while one was hand written, due to technical problems with the recorder. The interviews lasted on average for about 35 min (range 20–46 min). As soon as possible after an interview was completed, it was directly translated and transcribed to English. When nine interviews were completed, we assessed that we had sufficient information and had reached data saturation in relation to the aim of the study.

### Analysis

Discourse analysis was used [34]. In an initial analysis, all the transcripts were read multiple times in detail by the first and second authors. The next step involved performing a manual analysis focusing on discursive articulations [28] in the interview material. This means that we inductively examined how different ‘signs’ (key words, phrases and ideas) were linked to each other in various ways to produce different meanings. For example, in the caregivers’ accounts, young people were repeatedly linked to ideas of bad behavior, e.g. the youth group was regarded as being problematic. After examining similarities and differences between the recurring discursive articulations, eight discursive sub-themes were identified. Then after identifying how these themes related to each other, they were grouped and summarized into three main discourses. During the analytical process, the results were repeatedly compared with earlier interpretations and with the original transcripts in order to minimize any misinterpretation [35]. All authors made comments on the analysis and suggested revisions prior to reaching consensus on the interpretation of the interview material. The interviewees did not provide feedback on neither the transcripts nor the findings.

## Results

The three main discourses that were identified in the analysis of the caregivers’ narratives were: *Demoralized youths in a changing society; prevailing risks and modernity*, and *a generation gap.* These discourses are presented below as headings with subheadings representing the underlying discursive sub-themes (see also Figure 1). For the quotations, we use C = caregiver.

### Demoralized youths in a changing society

In this section, we will elaborate on a discourse identified in the interview narratives that is based on two coherent articulations (discursive sub-themes) - *youths as a problematic group*, and a *changing society*.

#### Youths as a problematic group

The interview narratives were characterized by articulations according to which young people were constructed as a problematic group cf. [36], and they were linked to ideas of being spoiled, demoralized, and corrupt, which reflected a change in society for the worse relative to earlier times. Thus, the caregivers often compared the conditions of today with how it was when they were young, in ‘the old days’. A general remark was that people were more generous and helpful before, and even if they lacked food themselves they still shared what they had with poor people. Thus, the narratives comprised nostalgic ideas of ‘the old days’ as something desirable, and they reinforced the assumed disparity between the past and the present [37].

The imagined differences between the societies of yesterday and today were also a question of the morals and the behaviors of young people. Several of the interviewees were worried about and disappointed with the lack of respect among children:

There are too many changes because years back it was much better and now even a 10 year [old] child is having a baby or is dating. Things are fast these days. […] They [today’s kids] are very stubborn when you talk to them and they are disrespectful, they don’t respect their caregivers and they dress the way they want (C7).

In this example, young people are described as different compared to how it was before; they are nowadays stubborn and without respect for their elders. Young women are regarded as dressing in improper ways, and young men as not taking responsibility in the way men did before, for example, when their girlfriends become pregnant.

#### A changing society

Thus, the caregivers not only attributed children with bad behavior in general, they also related a deterioration to a changing society. In other words, young people’s behavior was seen as a consequence of modern society. This tendency was reinforced by comparisons between traditional life and modern ‘Western’ life, with the latter usually regarded to be morally impoverished even if material conditions had improved. Some pointed out how the influence of western society in general has changed or replaced traditional ways of doing things by saying ‘These days everything is Western’ (C5), while others indicated even more profound changes. According to one caregiver, changes in food habits in today’s society have had concrete effects on children by making them mature and become fertile at an earlier age than before (C2). Another caregiver described similar tendencies when she said that ‘children are very hyper and they can get pregnant at the age of 10; it’s not like back in those days’ (C9).

Thus, young people of today are regarded to be both biologically mature and disrespectful, and this transformation is indirectly explained by a changing society. In other words, by describing a changing and more ‘dangerous’ society, the responsibility for young peoples’ behavior and moral decay was placed on surrounding circumstance and not on the caregivers or youths themselves. It was general tendencies in society at large that affected children and ‘transformed’ them into being disrespectful, and – as we will soon return to – the caregivers described themselves as almost helpless in relation to such tendencies, or maybe even as victims of structural forces. There were, in other words, traces of despair in their narratives.

### Prevailing risks and modernity

Against the background of ideas of a changing society and young people’s ‘bad‘ behavior, the interviewees identified some specific prevailing risks that threatened the youths (and especially girls). These risks were *early pregnancies*, *modern technologies*, and *STI/HIV –* and to some extent *contraceptives*. We will elaborate on how these can be seen as articulations of a discourse of risks, i.e. established ways to link many characteristics of modernity and young peoples’ lives to ideas of threats, dangers, and insecurity, even if some of them also include potentially positive aspects, such as modern technologies.

#### Early pregnancies

Several of the interviewees elaborated on the risks and problems that were related to becoming a parent at a young age. According to the caregivers, it was common that young girls dropped out of school – temporarily or permanently – when they became pregnant. One (C3) told about how his daughter, whom they had high hopes for, interrupted her first year of university studies because she got pregnant. Another caregiver (C5) indicated that early pregnancy – with both health risks and social risks – not only can result in dropping out of school, but can also be passed from one generation to the next: girls can repeat the behavior of their mothers and give birth at a young age.

The future of pregnant girls was also said to be ruined due to the behavior of ‘boys of today’ who were regarded as ignoring their responsibilities to the mother and the child:

… the boys of today have this thing of impregnating a girl and not supporting her, and I have seen it happen to me [i.e. my daughter and grandbaby] because that boy did not take responsibility for the baby. From the start they wanted each other, but he could not support his baby (C4).

Boys are thus considered to be a problem because they leave the girls (the mothers) to support themselves and the baby, and thereby risk the future of both the girl and the child. Single mothers are also considered to be a burden on their parents and their financial situation, especially when the father of the child ignores his economic responsibilities. The caregiver of the young mother is then forced to help out financially.

Thus, according to the discourse of risks, early pregnancies are threats not only against the social and economic future as well as health and wellbeing of young girls, but also for their caregivers. Furthermore, without education and work, young people risk becoming criminals, as one of the interviewees mentioned (C9).

#### Contraceptives and STI/HIV

Contraceptives and sexually transmitted infections including HIV (STI/HIV) were articulated directly or indirectly as contemporary threats against young people and their futures. However, especially when talking about contraceptives, interviewee narratives reflected conflicting interpretations. One interviewee said that while contraceptives are important to prevent early pregnancies and infections, they would also encourage girls to chase men (C2). STI/HIV was regarded to be a dangerous threat because it can be life threatening. However, the danger with HIV was usually not expressed explicitly, and it seemed to be taken for granted or was only mentioned in relation to other subjects. An example was when a caregiver talked about the risks with contraceptives (besides encouraging girls to chase men as was mentioned above):

… they have many different relationships with different kinds of men because she knows that she has injection [contraceptive] as a bodyguard. And by doing so she is inviting the STI/HIV because she doesn’t even know half of the men she is dating. We have given birth to different children and sometimes some are born with this disease and you realize it late that she has the disease and these days it’s easy to quickly see it [HIV] (C2).

In this case, contraceptives not only reinforce promiscuity, they also represent a threat against the mother as well as the newborn child because both can also be infected with HIV – which might be realized too late. Thus, contraceptives – as both good and bad – could both save and jeopardize the future of young girls and thus might undermine safe sexual behavior by providing a false sense of security and at the same time reinforce the awareness of safe sexual behavior [cf. 6].

#### Modern technologies

Modern technical devices and modern technologies were also articulated in terms of risks and threats because they were described as contributing to a decadent lifestyle. Mobile phones were one example, regarded as making it too easy to forge new contacts and because they lead young people into relationships. Their minds are on the phone, not on books, as one caregiver said (C1). Social media was also described as something bad:

I don’t know what to say, and I think it is the same [as] with the phone because they chat with people and start dating with people from... [Name of city] while they don’t even know each other. I don’t understand those things (C7).

In this case, the interviewee talked about the danger with modern technologies at the same time as she emphasized that she did not ‘understand those things’. However, the caregivers’ relationships to modern technologies were not only characterized by aversion and as unintelligible, but also by an explicit ambivalence in which they represented something on the one hand as good and important and on the other as bad and dangerous. The dual function of modern technologies was illustrated by cell phones. They were, as was mentioned above, considered to be a major threat because young people ‘even join the devil worship through these things’ (C6). But they could also be used in a positive way by caregivers to keep in contact with young persons. Another example of the ambivalence was television (TV). It was sometimes described as something morally bad because children watch dirty movies late at night making them ‘corrupt in their minds’ (C9). TV was occasionally also articulated in positive terms because it made young people stay at home in a safe environment, and - at best - learn new things. Thus, modern technologies and devices were regarded as having a positive effect as well as representing evil risks and threats against young people.

### A generation gap

In this section, we will elaborate on articulations about the *generation gap* and the interviewees’ notions about youths in contemporary society and how their narratives can be understood in terms of *ambivalence*, *respectability*, and a *morality of despair.*

#### Ambivalence

That the interview narratives were characterized by ambivalence can be understood in two ways. On the one hand, the ambivalence could be interpreted as an aspect of a risk discourse and as a source of confusion for the caregivers. If many major aspects of young people’s lives (social media, relationships, contraceptives, modern technologies, etc.) can be attributed different meanings, it can be difficult for caregivers to relate to these aspects and to guide young persons in their life choices. In other words, it can be a dilemma for caregivers how to communicate ideas of what can count as a preferable lifestyle; for example, is the use of contraceptives something good or bad, moral or immoral?

On the other hand, ambivalence could be described as a ‘communicative strategy’ where the caregivers used different and sometimes contradictory arguments with the common purpose of articulating a respectable lifestyle. The caregivers could, for example, describe the old days and traditional ways of living as morally superior in relation to the situation of today, but they could also use their own lives as warning examples with the purpose of motivating their children to higher studies and hence to hope for a better life. A woman took herself as an example of lost hope for the future, and she said that because she has no education and is dependent on her husband she cannot do what she wants to in life (C2). Thus, the ambivalence with the caregivers’ (seemingly) contradictory ways of relating to different phenomena was not just an expression of ambivalence per se, but was also a way of trying to impose a respectable lifestyle and a better future on young people.

#### Respectability

Besides the ambivalence, the interviewees’ rhetoric was characterized by strong ideas about how young people do behave and how they should behave, which can be described in terms of ‘respectability’. For example, by describing young people of today as stubborn and spoiled, and by linking early pregnancies to ideas of improper and risky sexual behavior, the caregivers indirectly constructed an ideal ‘image’ of how a respectable person should behave. To emphasize ideas of a respectable lifestyle is maybe not unusual among caregivers in general. However, a possible difference between these stories and the rhetoric of other caregivers in other social and cultural contexts is that these were constructed around some specific major risks such as HIV cf. [6] Thus, the lack of respectability is not only a risk of, for example, early pregnancies, but a risk that, according to the caregivers, can endanger the health, future, and life of young people and their descendants.

Respectability makes it important to behave properly and morally correctly and can also, in line with Skeggs [30], be regarded as a sign of social status. Respectability is in fact, according to Skeggs, usually the concern of those who are regarded by others to lack it, i.e. people with low socio-economic status. Respectability can represent the only resource that poor people have in the general social and cultural struggle to become someone, to have value, and to be taken seriously. This struggle can be directed towards higher socio-economic classes, but it is not unusual that it takes place within one’s own socio-economic group, drawing boundaries between different generations, genders, or occupations. The interviewees in this study live in a deprived rural area of SA and thus their striving for respectability might reflect not only an ambition to raise well-behaved youths, but also a socio-cultural strategy displaying a well-mannered family worthy of respect cf. [38].

#### Morality of despair

Taken together, ambivalence, the longing for respectability, ideas about the negative influences of modern society, and notions of risks, can all be linked to a position of despair. By this we mean that the interviewees’ morality-influenced statements in general were made against a background of feelings of despair, hopelessness, and inadequacy. The caregivers stated that young people behave in improper ways, but also that they are out of control and do as they please. In the end, the caregivers give up and just watch what happens:

But sometime it’s not about the family, it’s just that our child doesn’t care even if one want to know where are they going or where they have been and talk about that with her only to find that next week she have done the same and you end up giving up. You just watch her and see where it will end (C2).

Morality of despair can also be described as the result of a clash between the interviewees’ ambitions to nurture, educate, and discipline their children and their feelings of being helpless and powerless. This clash reflects a generation gap whereby it seems practically impossible for each generation to fully understand the other. The interviewees seemed almost desperate and without any possibilities to stand up against the forces of modernity and the western influences of today’s society. They explicitly stated that there was almost nothing they could do to guide young people on the right track besides talking to them: ‘I don’t see anything that can work except for talking’ (C9).

However, this was not the whole truth, because the interviewees also tried to give advice for how to help young people to lead a better life, but their efforts were – according to themselves – often without any significant result. One woman said that she has ‘given up on my boys’ (C6), and even if she was trying to guide them sexually by buying condoms, it still did not work because they did not use the condoms. Thus, the advice is often characterized by despair and a lack of power to influence the youths in a positive way.

## Discussion

The findings from this qualitative study illustrate that rural South African caregivers and their children and grandchildren are attributed different and sometimes opposing moral standards and different notions of proper sexuality. In other words, the interview material reflected a ‘clash’ between older and younger generations that is related to different ideas of what is regarded as a desirable lifestyle. Moreover, the interview narratives were characterized by ambivalence – an uncertainty about how different aspects of young peoples’ lives can be understood. This ambivalence reinforced the importance of perceptions of risks, making the entire life situation uncertain, and caregivers might also find modern society to be too multifaceted and feel that they have been left behind and that this is a reason and justification for avoiding communication about personal and sensitive issues with their children. The notions of ‘the modern society’ with all of its risks reinforced feelings of powerlessness related to ideas of immoral sexual behavior, and this is similar to what has been described in other African contexts. Dilger [39], for example, has shown how HIV functions as a metaphor for modernity and an immoral society, and even though cultural beliefs still have an important influence, globalization creates ambiguity between different messages regarding sexual behavior.

Feelings of insecurity, ambivalence and powerlessness, and a desire to win children’s respect, are – as mentioned before – not unusual among parents in general, but they can also be understood in relation to socioeconomic circumstances [30] and to a wider context, where SA’s political history of colonial and apartheid governance is of importance. While racial discrimination has been abolished in a legal sense and is not as evident as before, the historical legacy is still visible as socio-economic disparity between the country’s citizens [40].

Inequality is also clearly evident in rural areas where this study was conducted, in terms of poverty, unequal gender structures, and often poor access to health services [41]. The interviewees in our study lived in a poor rural area and for the most part lacked social, cultural, and economic capital. Rural citizens such as these seldom have resources to send their children to higher education, and unemployment and labor migration rates are high. Families are often shattered due to deaths related to HIV and to absent parents – especially fathers [42].

Against this poor sociocultural background, the interviewees’ feelings of despair and their desire for improved moral behavior become comprehensible. The only way to ‘become someone’ is to frame a high morality – to be respectable – or at least to communicate to the surrounding world that high morality is worth striving for (historically this has also been the case for western countries where lower socio-economic groups have used respectability as a means of reaching political and cultural legitimacy in relation to others) [43].

Respectability can also be understood from a gender perspective. Women in SA experience gender inequality with subordination and dependency, and have to deal with masculinities according to which polygamy is accepted in some communities and multiple sexual partners are common cf. [44]. Thus, by advocating a respectable lifestyle the caregivers reproduce a traditional morality, but they also offer a strategy for young women, and young people in general, to handle such masculine ideals. The quest for high morality also has a practical explanation because high morality represents a strategy in the efforts against STIs [39].

What, then, does this morality of despair mean for caregivers’ ability to function as role models and to transfer ideals of respectability to younger generations? As stated in the introduction, communication between parents and their children has been shown to have some positive effects when it comes to reducing risky sexual behavior among young people [45]. However, poor quality communication with adolescents probably has no effect on diminished sexual risk taking [46, 47], and while parents in some contexts feel quite comfortable discussing sexual matters, their children often do not [21]. When low levels of perceived parental monitoring have been recognized, a correlation between young people participating in risky health behaviors has been shown [48, 49].

The morality of despair described in our study is probably a poor foundation for communication between generations. Furthermore, the interviewed caregivers’ lack of economic, cultural, and social capital puts them in a situation without symbolic power and self-confidence, and as role models their position is weak. Their possibilities to guide or educate young people when it comes to issues concerning, for example, new technologies are limited, which means that young people turn to each other instead of relying on their caregivers [6]. Regarding sexuality and sexual health, the caregivers in our study explicitly stated that they try to influence their children, but with limited success. This has resulted in frustration, which was articulated in terms of immoral youths who disobey the older generation; such frustration has also been described in other contexts [22]. Communication problems between generations regarding sensitive matters such as sexuality and sexual health can also be related to the history of sub-Saharan Africa [50]. It has traditionally been more or less taboo for parents to discuss sexuality with their children. It is seldom that parents are involved in sexual socialization, and when they are, the involvement is often characterized by a one-way communication [23]. One explanation for these difficult dialogues might be the social hardship and migratory character of the South African labor force, and the many victims of HIV/AIDS where one parent, the grandparents, or others from the extended families take on the responsibility for raising the children [32].

Thus, the context of SA – with its history of apartheid, gender structures, changing traditional norms, HIV, and poverty – provides an explanation for the morality of despair and the interviewees’ feelings of powerlessness and inadequacy. However, this morality cannot be regarded as only an effect of historical circumstances, it is also an active strategy from the caregivers’ perspectives to create a respectable life and to protect young people from risks. It seems, however, as if the morality of despair is a counterproductive position because it is the expression of the caregivers’ lack of symbolic capital and hence the lack of power to directly influence young people and hence amplify the generation gap.

One limitation of this study is that it does not clarify how morality of despair actually influences the interaction between caregivers and young people in practice, especially as the relation between discourses and actual behavior is complex cf. [51]. Thus, it is difficult to state that the discourses and the moral regime identified in the interview material would necessarily comprise the same meanings if we have had studied how caregivers and young people communicate in everyday settings. Other or complementary methods such as participant observations might be one possible strategy in future research that could address this limitation.

### Implications for practice

Regarding transferability, the findings report on dialogues between nine caregivers and the interviewers. However, even in smaller studies, each interview is viewed as mirroring broader practices and discourses in society [33, 52]. In view of this, we believe that the findings from this study in Bushbuckridge might be transferable to other rural and disadvantaged contexts in SA as well as to low-income countries with similar socio-economic and cultural settings, especially in Sub-Saharan Africa. More importantly, the findings may hopefully initiate important discussions regarding how to interpret and solve communication difficulties between generations.

When taking the findings of this exploratory study into consideration, it is difficult to single out a specific approach to addressing caregivers’ ‘morality of despair’ when they attempt to impose traditional morals and a respectable lifestyle in young people. However, it is important to emphasize the importance of communication across generational boundaries and of interventions based on knowledge about local cultural norms in both the older and younger generations.

Previous studies have suggested the importance of including and empowering caregivers and other adults in efforts to improve their courage and skills and thus to increase their ability to responsively meet young people’s need for knowledge about sex and prevailing risk-taking in general [20, 52, 53]. Although caregivers are very important, sexual education does not work unless adults not only have the knowledge, but also are comfortable and feel able to have a two-way communication about sensitive issues [46, 47]. Moreover, when caregivers want to talk about sexuality and risks with their children or grandchildren, it is important that they are open and understanding, and not just agitate about the importance of sexual abstinence. Young people may also need other adult mentors to complement their parents [54].

An understanding of shared local context and cultural norms are required in developing appropriate interventions with information the younger generation really asks for and requires [55–58]. Sexual education at school might miss out on what young people really need to know about sex and gender dynamics within their local contexts. Furthermore, schools might overlook that young people also want to talk about being in love and feeling pleasure, not only about risks [17]. It can also be the case that young people are fully aware of the risks associated with particular sexual behaviors but also trivialize the risks, and embrace feelings of romance and coexisting concepts steered by local traditions, normative gendered behavior and peer pressure [6]. Thus, interventions including sexual education have to treat young peoples’ sexuality as a multifaceted phenomenon, and to take local cultural norms, overall gender patterns, and the will of young people themselves into consideration [59].

## Conclusion

The findings from this study add to the body of earlier research about obstacles to intergenerational communication e.g .[18–25]. Three central discourses identified in the interview narratives were: *demoralized youths in a changing society, prevailing risks and modernity and a generation gap*. According to these discourses, young people were seen as a problematic group in a changing society. Adolescents were mainly associated with risks related to early pregnancies, modern technologies, HIV/STI and contraceptives. The interviews also showed that the caregivers wanted to impose their ideas of a “respectable” lifestyle on young people. However, they were frustrated because of differences in perception of life values between their own and the younger generation. This perceived gap counteracted their abilities to guide and to communicate with young persons. To overcome adults’ ‘morality of despair’ with feelings of ambivalence, hopelessness and lack of power, we recommend further research about how a broad and well-founded national and community collaboration linked to school-based programs can support family participation in order to empower adults’ communication with young people regarding a ‘respectable’ lifestyle, sexual health, and risk-taking [20, 60, 61].

## Supplementary information

**Additional file 1.**

**Additional file 2.**

## Data Availability

The datasets used and/or analysed during the current study are available from the corresponding author on reasonable request.

## Abbreviations

STI: Sexually Transmitted Infections
HIV: Human Immunodeficiency Virus
SA: South Africa
HDSS: Health and Socio-demographic Surveillance System
C: Caregiver
