# Supplementary material for: Obstacles to intergenerational communication in caregivers’ narratives regarding young people’s sexual and reproductive health and lifestyle in rural South Africa
Source: BMC Public Health. 2020 May 27;20:791. doi: 10.1186/s12889-020-08780-9 (PMC7251858; doi:10.1186/s12889-020-08780-9)
Supplement: Supplementary file 1 — Additional file 1. [file 12889_2020_8780_MOESM1_ESM.docx]

Figure 1. The obstacles to intergenerational communication presented from the study results as three main discourses and eight discursive sub-themes.
