# Supplementary material for: Obstacles to intergenerational communication in caregivers’ narratives regarding young people’s sexual and reproductive health and lifestyle in rural South Africa
Source: BMC Public Health. 2020 May 27;20:791. doi: 10.1186/s12889-020-08780-9 (PMC7251858; doi:10.1186/s12889-020-08780-9)
Supplement: Supplementary file 2 — Additional file 2. [file 12889_2020_8780_MOESM2_ESM.docx]

| **INTERVIEWS WITH PARENTS/GUARDIANS** to young men and women (18-19)  *Remember, to not push and to say that* *there are no right or wrong answers, do not judge* | |
| --- | --- |
| - **Start the recording by saying the date and the code # of the interview** | |
| **Main questions** | *Elaborative questions* |
| 1. **What do you think it means when adolescent girls become pregnant while still in school?** | - - *For herself*   - *For her education and future?*   - *For her family?* |
| 1. **When do you think is the ideal age of getting pregnant?** | - - *How old where you and your wife/husband when you had your first baby?*   - *Was that a good age?* |
| 1. **What do you think can be done to prevent teenage pregnancies?** | - - *Is that an important thing to do?* |
| 1. **What can be done to support pregnant adolescent girls?** |  |
| 1. **What about boys’ responsibilities?** | - - *Do they get off lightly?*   - *What about the boys’ family?* |
| 1. **What is the situation for adolescent boys and girls today compared to earlier, let’s say 5-10 years ago?** | - - *What about risks?*   - *Changes in the society?*   - *Other changes?* |
| 1. **Do media like internet, magazines, TV, cell-phones etc. have an influence on adolescent boys and girls?** | - - *In a bad or good way or both? Please describe*   - *For your teenager?* |
| 1. **Do you want to add something?** | - - *Forgot to say something?* |
| 1. **How did you feel during the interview?** |  |
